# Vinyl Chloride Degradation Using Ozone-Based Advanced Oxidation Processes: Bridging Groundwater Treatment and Machine Learning for Smarter Solutions

**DOI:** 10.3390/molecules30244737

**Published:** 2025-12-11

**Authors:** Jelena Molnar Jazić, Marko Arsenović, Tajana Simetić, Slaven Tenodi, Marijana Kragulj Isakovski, Aleksandra Tubić, Jasmina Agbaba

**Affiliations:** 1 Department of Chemistry, Biochemistry and Environmental Protection, Faculty of Sciences, University of Novi Sad, Trg Dositeja Obradovića 3, 21000 Novi Sad, Serbia; tajana.djurkic@dh.uns.ac.rs (T.S.); slaven.tenodi@dh.uns.ac.rs (S.T.); marijana.kragulj@dh.uns.ac.rs (M.K.I.); aleksandra.tubic@dh.uns.ac.rs (A.T.); jasmina.agbaba@dh.uns.ac.rs (J.A.); 2 Faculty of Technical Sciences, University of Novi Sad, Trg Dositeja Obradovića 6, 21000 Novi Sad, Serbia

**Keywords:** vinyl chloride, ozone-based AOPs, groundwater treatment, artificial intelligence, advanced machine learning models

## Abstract

Water scarcity is fostering an urgent need to drive research into novel and synergistic water treatment approaches, with advanced oxidation processes (AOPs) emerging as a superior option for treating various contaminants. The spread of vinyl chloride (VC) through groundwater sources raises concerns for potable water production due to its toxic and carcinogenic properties. This study integrates ozone-based degradation experiments with data-driven modelling approaches to statistically characterize and predict VC removal under different water-matrix conditions. Ozonation alone enables partial removal of VC from two contaminated groundwater samples, while integration of O_3_/H_2_O_2_ treatment further enhances the degradation efficacy (70–97%). Decreasing VC concentration below the parametric value of 0.5 µg/L requires application of the peroxone process or photodegradation by O_3_/H_2_O_2_/UV for groundwater with higher levels of interfering compounds. Advanced machine learning models and ensemble methods were also tested to enhance predictive accuracy for target molecule degradation, considering water characteristics and treatment parameters as input features. An ensemble of Random Forest and Neural Network predictions yielded the best performance (R^2^ = 0.99; Mean Squared Error = 10.8), demonstrating the effectiveness of ensemble approaches for complex chemical prediction tasks and highlighting areas for further refinement to improve interpretability and predictive consistency of AOP treatment outcomes. This study not only aligns with the current momentum in AI-assisted AOP research but also advances it by delivering a generalizable, reproducible, and interpretable ensemble model trained on experimentally diverse datasets.

## 1. Introduction

Contaminated groundwater represents an urgent environmental issue, especially considering its frequent use as a vital resource for water supply. Chlorinated hydrocarbons, including vinyl chloride, trichloroethylene, tetrachloroethylene, chloroform, carbon tetrachloride, and chlorobenzenes, are among the most critical toxic organic pollutants found in groundwater [1]. Chlorinated hydrocarbons are commonly used as key raw materials and organic solvents in industry, including in the production of pesticides, pharmaceuticals, leather, and dyes. The spread of these contaminants through soil and groundwater sources is caused by improper handling, storage, and application in chemical industrial zones, mining areas and other polluted sites worldwide [2].

Vinyl chloride typically originates from industrial facilities that produce polyvinyl chloride, hazardous waste sites, and landfills as a byproduct of the biodegradation of chlorinated organics. Vinyl chloride, accompanied by 1,1-dichloroethene, are by-products of the anaerobic biodegradation of perchloroethene, trichloroethene [3] and 1,1,1-trichloroethane [4]. These volatile daughter compounds are less biodegradable, more toxic, and more mobile than their parent compounds, tending to accumulate in contaminated aquifers where they pose greater environmental risks [4]. In situ remediation of groundwater contaminated with complex chlorinated hydrocarbon molecules can be effectively implemented through reductive electrochemical technology under controlled conditions [1] or carbon materials such as biochar and modified zero-valent iron [5,6].

Due to its extreme toxicity and carcinogenicity to humans, Council Directive 2020/2184 [7] and 98/83/EC [8] of the European Union established the limit for vinyl chloride levels in drinking water at 0.5 μg/L. To meet this standard, ozonation is frequently utilized in drinking water treatment plants as an abatement technology to degrade chlorinated volatile compounds from groundwater intended for potable use. The oxidation of chlorinated ethenes by ozone is highly selective with electron-rich moieties, and the process efficacy decreases as the degree of chlorination increases [3,8]. Ozone is also depleted in reactions with inorganic substances such as iron, manganese, and ammonia nitrogen, facilitating their removal from water [9].

To enhance the efficiency of ozone oxidation, advanced oxidation processes (AOPs) leverage the high and non-selective oxidation potential of hydroxyl radicals and other reactive oxygen species (ROS), providing new avenues for degrading a wide range of organic pollutants [8,9,10]. Within the framework of AOPs, various approaches can be used to improve ozone oxidation, including the simultaneous or subsequent addition of hydrogen peroxide, solid catalysts with semiconductor properties, or UV irradiation [9,10]. Previous studies have focused on the degradation of chlorinated ethenes by ozone [3], ozone and hydrogen peroxide kinetic study in distilled water [11,12], a TiO_2_/UV/O_3_ system [13], photochemically induced reaction of ozone in argon matrixes using irradiation with red LED (625 nm) and UV (254 nm) light [14], UV/H_2_O_2_ [15], and *Ultrox system* UV/O_3_/H_2_O_2_ [16] processes. Among the limited number of available published studies, only a few have addressed vinyl chloride degradation in real groundwater [13,15,16] and insufficient attention has been paid to the combined effects of water matrix composition, ozone-based AOP mechanisms, and utilising predictive modeling under realistic conditions, which limits their practical applicability.

Recently, the incorporation of artificial intelligence (AI) approaches has emerged as a complementary analytical tool in the water and wastewater treatment sector [17,18]. Machine learning refers to statistical modelling approaches that learn patterns from data and generate predictive outputs without explicit programming [19]. State-of-the-art AI and deep learning techniques can be applied to overcome limitations encountered throughout water treatment including chemical and separation technologies, addressing data scarcity, optimizing treatment parameters, and enhancing predictive capabilities. Previous research indicates that the application of AI approaches has improved various aspects, including the prediction of coagulant doses, extending the application of AI to socio-economic dimensions [20], membrane separation [21,22], the degradation of micropollutants [18], and the formation of adverse disinfection byproducts during disinfection [23]. In the field of chemical treatment by AOPs, AI neural network models have been used to optimize materials, conditions, and pollutant removal by catalytic oxidation, catalytic wet air oxidation, and electrochemical oxidation [24]; applied in UV/H_2_O_2_ and ozonation applications for pharmaceutical degradation, achieving significant improvements in treatment efficiency through parameter tuning [25]; to predict total organic carbon removal, underscoring the value of data-driven approaches under varied operational scenarios [26], and to guide the development of catalysts using machine learning [27]. Recent studies have highlighted the growing role of AI in supporting the design and optimization of AOPs across various water treatment contexts. However, most existing studies rely on simplified conditions and provide limited insight into real groundwater systems, indicating the need for a more comprehensive and application-oriented approach.

Despite the growing emphasis on AI solutions in (waste)water treatment, water quality protection, and process control, to the best of our knowledge, there is no available data regarding the application of AI models to foster degradation studies of chlorinated organic contaminants by ozone and AOPs. This study aims to extensively investigate the degradation of vinyl chloride from contaminated groundwater by ozone and ozone-based AOPs, focusing on the impact of water matrix and oxidation by-products formation. AI has been utilized to boost the integration of ozonation process with various approaches to initiate the generation of reactive free radicals, and moreover, to enhance the accuracy of predicting vinyl chloride degradation using advanced machine learning models and ensemble methods.

## 2. Results and Discussion

### 2.1. Effect of Ozonation and Ozone-Based AOPs on Vinyl Chloride Degradation

**Figure 1:**
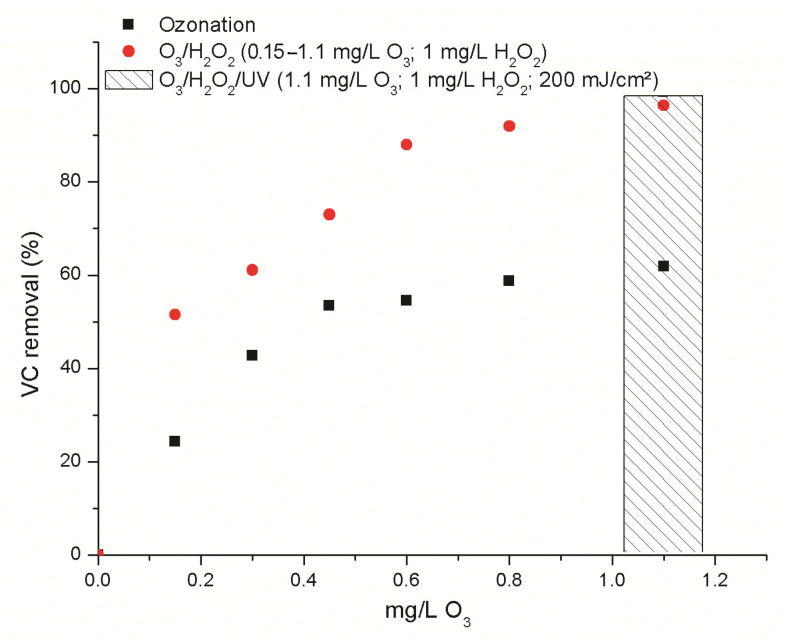
Removal of VC from GW1 by ozonation and ozone–based AOPs.

**Figure 2:**
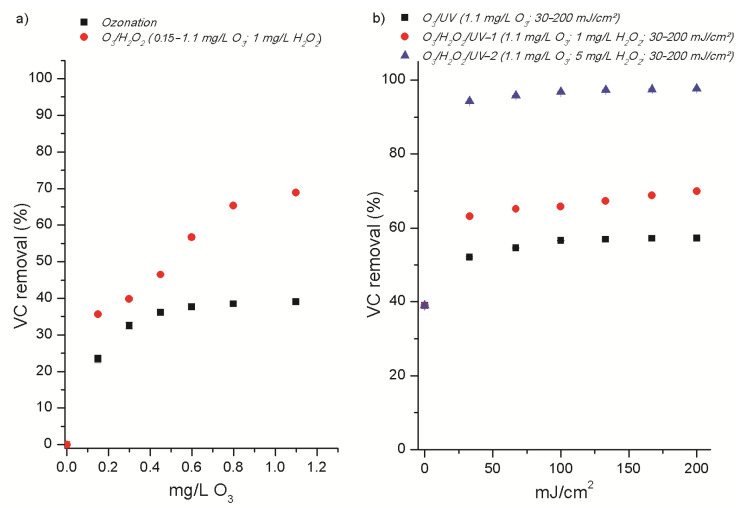
Removal of VC from GW2 by: (**a**) ozonation and O_3_/H_2_O_2_ processes, and (**b**) photochemical ozone-based AOPs.

The results of vinyl chloride degradation in synthetic and natural water matrices (as described in Section 3.2) by the ozonation process are presented in Figure S1, Figure 1 and Figure 2. The removal of vinyl chloride was significantly more effective in the synthetic water matrix, in the absence of interfering ions, compared to the treatment of real groundwater (GW1 and GW2). Ozone treatment (0.2–0.3 mg/L O_3_) of synthetic water enriched with vinyl chloride (25 ± 0.5 µg/L) enables the reduction of vinyl chloride concentration to below the limit value of 0.5 µg/L.

Vinyl chloride removal by the ozonation process was significantly inhibited in groundwater, with a removal of approximately 60% acheived in GW1. The application of the peroxone process (1.1 mg/L O_3_; 1 mg/L H_2_O_2_) and the O_3_/H_2_O_2_ process with subsequent UV treatment (1.1 mg/L O_3_; 1 mg/L H_2_O_2_; 200 mJ/cm^2^) improved VC removals, reaching concentrations as low as 0.43 ± 0.05 µg/L and 0.34 ± 0.02 µg/L, respectively. Progressive removal of vinyl chloride was observed in the concentration range 0.2–0.6 mg/L O_3_, with no significant change in treatment efficacy as the ozone dose increased further (Figure 1). Degradation of vinyl chloride by the ozonation process (0.2–1.1 mg/L O_3_) in GW2 was even further suppressed, achieving a maximum removal of 40%. The addition of hydrogen peroxide in the peroxone process results in significant improvements over ozonation alone, enabling 70% removal of VC. During the peroxone process, ozone decomposition is initiated by the hydroperoxide anion, subsequently leading to a synergistic effect between ozone and hydrogen peroxide and resulting in the production of hydroxyl radicals. The complex reaction chain mechanism of the peroxone process is presented in the Supplementary Material [28].

The control UV treatment alone (UV fluence 30–200 mJ/cm^2^) had a negligible effect on VC removal (changes below 5%) in both groundwater samples. Although the subsequent UV treatment (O_3_/UV) significantly enhanced VC removal from GW2 against ozonation alone, the UV treatment did not further improve the efficacy of peroxone (O_3_/H_2_O_2_/UV). Alternatively, the addition of H_2_O_2_ to ozonated water just prior to UV irradiation increased VC removal (70–98%). The higher concentration of H_2_O_2_ had a notable effect on improving the process efficiency, while no significant influence was observed when increasing UV fluence in the range of 40–200 mJ/cm^2^, likely due to oxidant decomposition and consumption of ROS, limiting further radical formation (Figure 2). Within the framework of the target molecule’s degradation mechanism, photolysis of residual ozone in water might generate hydrogen peroxide as an intermediate and molecular oxygen, which further initiates the formation of HO**∙** under UV irradiation. The reaction between ozone and hydrogen peroxide (peroxone process) is also possible but proceeds at a significantly lower rate. Photolysis of hydrogen peroxide leads to HO**∙** formation. In addition, hydrogen peroxide undergoes acid–base equilibrium (pKa = 11.7), where the conjugate base (HO_2_^−^) can also participate in the radical chain mechanism. Supporting reactions are provided in the Supplementary Material. Overall, the radicals formed through these processes contribute to the degradation of vinyl chloride in water during the application of ozone-based AOPs.

The oxidation study by Von Gunten [12] reported that VC reacts rapidly with ozone, with a half-life of 2.5 s for a concentration of 1 mg/L O_3_, which aligns with our findings for the synthetic water matrix. Formaldehyde, formyl chloride, and formic peracid are the major products of the oxidation of vinyl chloride and trans-1,2-dichloroethene with ozone [12]. The significantly depressed groundwater treatment efficiency is driven by the substantial inhibitory effects of iron, manganese, ammonia, and natural organic matter, which consume ozone and ROS for their oxidation. Kokkoli et al. [3] revealed that treatment of chlorinated ethenes-contaminated groundwater by ozonation (0.23 g O_3_/m^3^) allowed more than 90% removal of vinyl chloride and trans-dichloroethene (DCE), while the degradation of cis-DCE, 1,1-DCE, and trichloroethene required higher ozone doses. They demonstrated a beneficial synergy between ozonation at low ozone concentrations and subsequent GAC filtration for the removal of chlorinated ethenes from groundwater. The available data concerning the photochemical reactions of ozone and monohaloethenes are scarce. Ault [14] indicates that the reaction of ozone with vinyl chloride in argon matrices is wavelength-dependent, leading to the formation of products including ketene, chloroketene, HCl, and presumably H_2_. Dutschke et al. [13] report a carrier-bound TiO_2_/UV/O_3_ catalytic laboratory treatment of contaminated groundwater achieving degradation rates of cis-dichloroethene, trichloroethene, and tetrachloroethene of up to 98%, and 85% for trichloromethane. The degradation of chlorinated ethenes by hydroxyl radicals is significantly hindered at higher chlorination levels of the ethene derivates.

**Figure 3:**
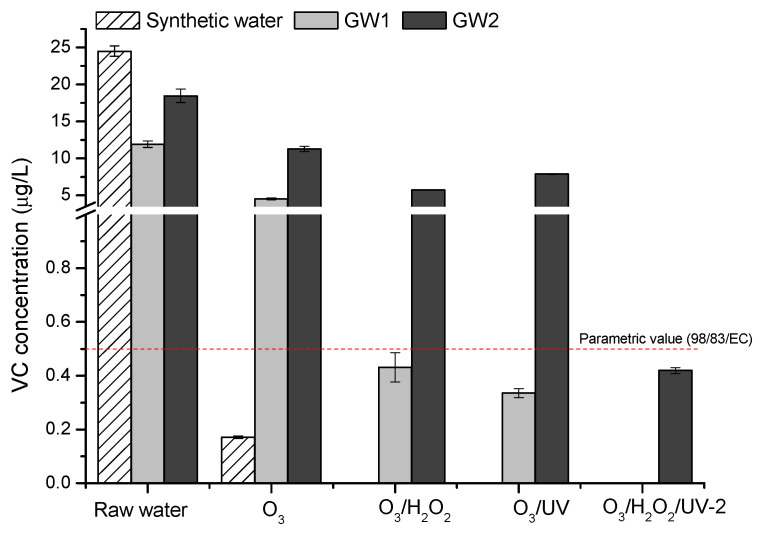
Residual VC concentration in ozone- and AOPs-treated groundwater: O_3_ [1.1 mg/L O_3_]; O_3_/H_2_O_2_ [1.1 mg/L O_3_; 1 mg/L H_2_O_2_]; O_3_/UV [1.1 mg/L O_3_; 200 mJ/cm^2^]; O_3_/H_2_O_2_/UV-2 [1.1 mg/L O_3_; 5 mg/L H_2_O_2_; 200 mJ/cm^2^].

Based on the literature data and our investigation (Section 2.2), it can be assumed that in ozone-based AOPs, the degradation of vinyl chloride proceeds via the Criegee mechanism, and cleaves into a carbonyl compound and a hydroxyhydroperoxide, with major degradation intermediates such as formaldehyde, formic peracid, and formyl chloride (which may further decompose into CO and HCl), as presented by Dowideit and von Sonntag [29]. Furthermore, our study demonstrates that the application of ozone alone is incapable of reaching the parametric value for VC during groundwater treatment. In order to decrease VC concentration below limit value of 0.5 µg/L, the peroxone process is required for GW1 and the O_3_/H_2_O_2_/UV process for GW2, applying a higher hydrogen peroxide concentration prior to UV treatment (Figure 3). In summary, for both water matrices the optimal O_3_/H_2_O_2_ mass ratio for the peroxone process was approximately 1:1, while for GW2 treated by the O_3_/H_2_O_2_/UV process the optimal oxidant ratio was 1:0.2 (based on initial ozone concentration), indicating faster oxidant decomposition under the subsequent UV treatment.

### 2.2. Effect of Ozonation and Ozone-Based AOPs on Oxidation By-Products Formation

Our research indicates that the water matrix exhibits a strong inhibitory effect on VC removal by ozonation and ozone-based AOPs. Ozone and ROS formed during advanced oxidation are often consumed in reactions with natural organic matter and certain inorganic species. The higher efficiency of ozonation and ozone-based AOPs observed for synthetic water and GW1 compared to GW2 is likely due to the higher content of iron and manganese, as well as organic matter that interacts with ozone and oxidative species.

**Figure 4:**
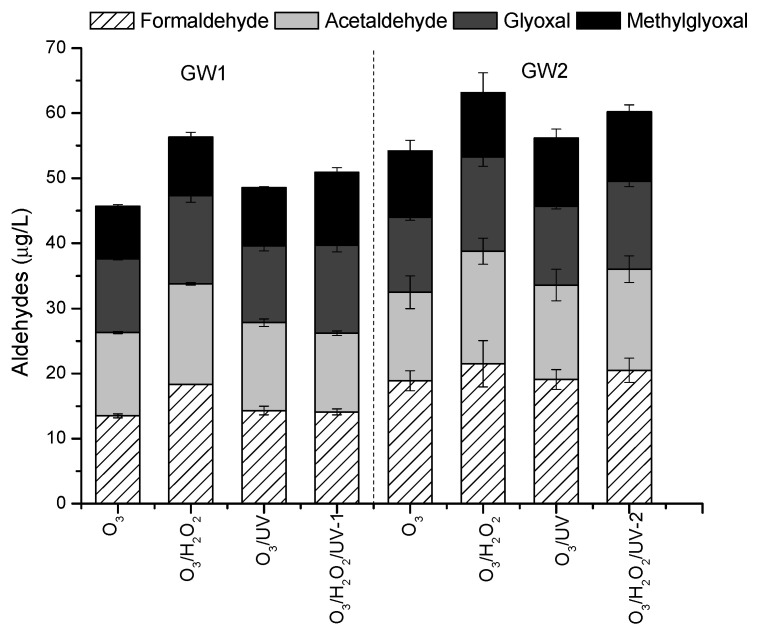
Effects of ozonation and ozone-based AOPs on the formation of aldehydes in water.

The water characteristics after oxidation treatments are presented in Table S1. In the treated water, the iron concentration decreased more significantly than the manganese concentration. The content of organic matter, based on the TOC value, was reduced by 22–28% during ozonation and ozone-based AOPs. As a result of the oxidative transformation of organic matter present in groundwater, an increase in the concentration of aldehydes was recorded, as oxidation by-products of ozone, accompanied by hydroxyl radicals (Figure 4, Table S1).

Oxidation treatments led to a seven-fold increase in aldehyde content compared to the raw water, with slightly higher concentrations measured in water treated by the peroxone process (56.3–63.2 µg/L). In water treated by ozonation and ozone-based AOPs, acetaldehyde and formaldehyde were slightly more prevalent than glyoxal and methylglyoxal. Formaldehyde may also be formed as an intermediate of VC degradation. WHO (2011) [30] data indicate that formaldehyde concentrations in ozonated drinking water can reach levels up to 30 µg/L. Given the significant disparity between anticipated formaldehyde concentrations in drinking water and the tolerable limit, a formal guideline value has not been set (the previous edition included a guideline value of 900 µg/L). Current data suggest that formaldehyde is not carcinogenic through the oral route [30]. Oxidation of organic matter by ozone and related processes increase their biodegradability. However, the resulting carbonyl compounds can be significantly reduced by subsequent granular activated carbon filtration [31]. The formation of bromate in the ozone- and AOP-treated waters was not observed, which aligns with the low bromide content in the groundwater samples. Maintaining a low level of bromate (below 10 µg/L) in drinking water is of paramount importance due to its poor removal by granular activated carbon and health concerns [31]. Guideline values have not been established for other possible VC degradation intermediates in drinking water [30].

### 2.3. Artificial Intelligence Models for Predicting Vinyl Chloride Degradation: Performance, Interpretability, and Error Analysis

The following models were trained and evaluated: Linear Regression, Ridge Regression (L2 regularization), Random Forest, Gradient Boosting, and a Multi-Layer Perceptron (Neural Network). In addition, a Stacked Ensemble combining these models was implemented to assess potential synergistic performance. All models were evaluated on a held-out 30% test set using coefficient of determination (R^2^), mean squared error (MSE) and mean absolute error (MAE) as metrics. The results are summarized in Table 1. The performance across models reveals valuable insights. Linear and Ridge regressions demonstrate high explanatory power (R^2^ > 0.95), suggesting that a considerable portion of the variance in VC degradation can be captured through linearly separable descriptors when carefully engineered. However, Random Forest outperforms all other models, confirming its capability to capture intricate nonlinear interactions between water matrix components and AOP parameters. The ensemble model, while slightly less accurate than Random Forest, offers conceptual value through the combination of generalization and regularization mechanisms embedded in neural, tree-based, and linear estimators. All models, including Linear Regression, were trained on the same full set of raw and engineered features; therefore, the lower performance of linear models reflects their limited ability to represent nonlinear interactions inherent to AOPs rather than any difference in dataset size or variable selection.

**Figure 5:**
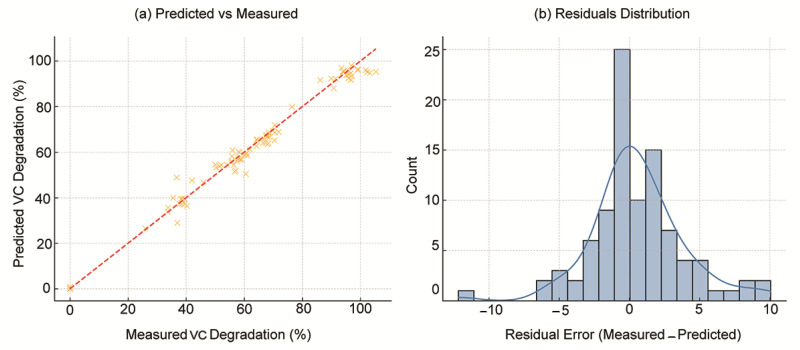
(**a**) Predicted vs. measured VC degradation (%) on the held–out 30% test set (n = 31); (**b**) Residual error distribution for the same test set.

To evaluate the predictive precision of the models beyond summary metrics, graphical analyses were performed. Figure 5a presents a scatter plot of predicted versus experimentally measured VC degradation values. The majority of predictions cluster along the ideal diagonal line, indicating high accuracy and low bias. Furthermore, Figure 5b displays the distribution of residuals (difference between true and predicted values). The distribution is centered near zero, with minimal skewness and no prominent outliers, confirming well-behaved model errors.

**Figure 6:**
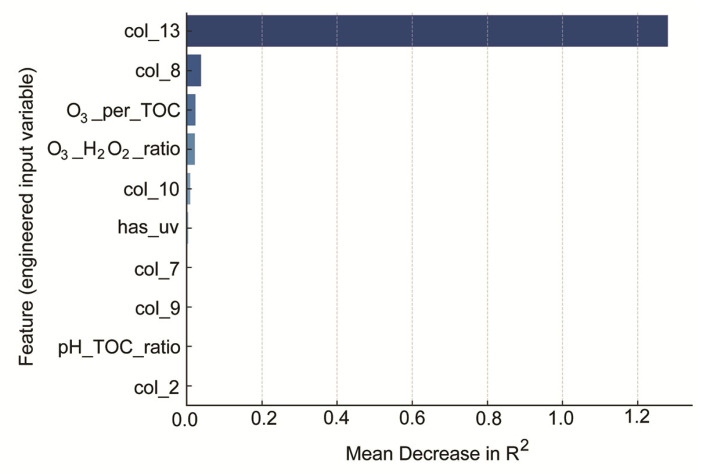
Permutation feature importance (Top 10) for the Random Forest model.

Model interpretability was assessed using permutation importance, as shown in Figure 6 [5]. The y-axis in Figure 6 lists the top 10 input features ranked by their contribution to predictive performance. These features include both raw experimental variables and engineered descriptors. For clarity, variable names such as col_13, col_8, and col_10 correspond to ozone concentration, hydrogen peroxide concentration, and UV fluence, respectively. Other labels, such as O_3__H_2_O_2__ratio, O_3__per_TOC, and pH_TOC_ratio, represent normalized ratios derived from domain-relevant chemical relationships. The full mapping of feature names to their physical meaning is provided in the supplementary description. The x-axis represents the mean decrease in model R^2^ caused by randomly shuffling each feature’s values. This method quantifies the importance of each variable by measuring the extent to which it contributes to predictive accuracy. As shown, the most influential predictors included ozone dose, hydrogen peroxide dose, the ozone-to-H_2_O_2_ ratio, TOC-normalized exposure to ozone, and UV-related indicators. These results align well with known mechanisms of AOP efficiency and further support the inclusion of engineered descriptors in data-driven modeling. A limitation of the present modelling approach is the restricted number of groundwater matrices. Although dataset augmentation increased numerical robustness, broader sampling across aquifers with differing geochemical properties would further enhance model generalizability. Thus, further research will extend to ML-based optimization of O_3_/H_2_O_2_/UV ratios using a substantially broader range of groundwater samples and a broader experimental dataset to prevent overfitting and ensure reliable extrapolation.

To the best of our knowledge, this is the first study that employs advanced AI models for predicting VC degradation, based on experimental results obtained from two groundwaters and a synthetic water matrix subjected to AOP treatments. Compared to previous studies, our study offers a unique combination of methodological rigor and practical relevance. Specifically, we applied a multi-model ensemble framework-combining tree-based, linear, and neural learners-to predict the degradation of VC, a chlorinated organic contaminant of regulatory concern. Unlike previous studies that rely on synthetic datasets or single-source samples, our models were trained on experimental data collected from two real groundwater sources and one synthetic water matrix, thus covering a broader range of physicochemical conditions. In addition, through data augmentation, feature engineering, and interpretability analysis (e.g., permutation importance), we provide a robust and transparent modeling approach.

While previous studies have reported promising predictive performance, many do not provide systematic comparisons across multiple algorithms or real-world datasets. For instance, Serna-Carrizales et al. [25] achieved R^2^ values ranging between 0.91 and 0.94 using feed-forward neural networks trained on AOPs applied to pharmaceutical residues. Zhang et al. [26] reported R^2^ ≈ 0.95 for reaction outcome prediction, though their models were calibrated solely on synthetic water matrices. In contrast, our ensemble-based model achieved a higher predictive accuracy with R^2^ = 0.987 and MSE = 10.78, representing one of the most accurate VC degradation models reported to date.

Furthermore, only a few of these studies [23,27] provide insight into model interpretability or error diagnostics. In our work, residual analysis and permutation-based feature importance were incorporated to assess both performance and transparency, reinforcing the credibility of our modeling pipeline. This level of analysis is essential for practical implementation in environmental systems, where explainability is often a prerequisite for regulatory acceptance and operational integration.

## 3. Materials and Methods

### 3.1. Chemicals and Reagents

The analytical standard of vinyl chloride used in this study was purchased from Dr. Ehrenstorfer™ (M20.1, 1000 µg/mL in Methanol, CAS no. 75-01-4, Augsburg, Germany). Formaldehyde (Fluka, CAS no. 50-00-0, Buchs, Switzerland), acetaldehyde (Sigma-Aldrich, CAS no. 75-07-0, St. Louis, MO, USA), glyoxal (Acros Organics™, CAS No. 107-22-2), and methylglyoxal (Acros Organics™, CAS no. 78-98-8, Geel, Belgium) were used for aldehydes analysis. Potassium indigotrisulfonate (Sigma-Aldrich, CAS no. 67627-18-3, St. Louis, MO, USA) was used as an ozone-scavenging reagent. Hydrogen peroxide (30% *w*/*w* H_2_O_2_, POCH, Gliwice, Poland, CAS No. 7722-84-1) was applied in O_3_/H_2_O_2_ and O_3_/H_2_O_2_/UV treatments. Methanol (CAS No. 67-56-1) and hexane (CAS No. 110-54-3), ultra resi-analyzed^®^ grade, were purchased from J.T. Baker^®^ (Phillipsburg, NJ, USA). Ultrapure water (ASTM Type I quality) was obtained from a LABCONCO (Water Pro RO/PS Station, Kansas City, MO, USA) system (water of American Society for Testing and Materials type I quality) and used for the preparation of reagents. All other chemicals were analytical grade and were used without further purification.

### 3.2. Water Samples

Groundwater contaminated with vinyl chloride was sampled at two locations from piezometers and used for this investigation (GW1 and GW2). The characteristics of the groundwater samples are provided in Table 2. A synthetic water matrix was prepared using ultrapure water spiked with VC to achieve a final concentration of approximately 25 μg/L.

### 3.3. Ozonation and Ozone-Based AOPs

The treatment of groundwater by ozonation and ozone-based AOPs was investigated under laboratory conditions to simulate “pump-and-treat” technologies operating at the existing pH. Ozonation and O_3_/H_2_O_2_ treatments were conducted in a 6 L glass column. Ozone gas, produced from ambient air using an Ozomax generator (Ozomax Ltd., Granby, QC, Canada), was injected into the water through a diffuser placed at the bottom of the column. The ozone generator system contained an air compressor, air dryer, and molecular sieves. The transferred ozone doses applied ranged from 0.1 to 1.1 mg O_3_/L, based on the ozone demand. The O_3_/H_2_O_2_ (peroxone process) was investigated by dosing a freshly prepared hydrogen peroxide solution into the water directly before ozonation. The H_2_O_2_ concentration applied was 1 mg/L, corresponding to a mass ratio of 0.15–1.1 O_3_ relative to H_2_O_2_.

Photochemical AOPs were conducted in a 700 mL reactor, equipped with a low-pressure lamp (16 W TUV Philips, Amsterdam, The Netherlands) emitting monochromatic radiation at 253.7 nm, as described by Simetić et al. (2024) [32]. For the photochemical AOPs treatment, water ozonated by 1.1 mg O_3_/L was withdrawn from the glass column and immediately subjected to subsequent UV treatment (O_3_/UV) or combined with the dosing of hydrogen peroxide just prior to UV treatment (O_3_/H_2_O_2_/UV). The residual ozone in the water exposed to the UV treatment was 0.15–0.2 mg/L, the applied UV fluence ranged from 30 to 200 mJ/cm^2^, while the applied concentration of H_2_O_2_ for the O_3_/H_2_O_2_/UV process was 1 mg/L. All experiments were conducted at least in duplicate, at a temperature of 18–22 °C. The schematic diagram of the water treatment is shown in Figure S2.

### 3.4. Analytical Methods

Vinyl chloride in the water samples was directly analyzed with a purge and trap system (Lumin 15-2500-074, Teledyne Tekmar, Mason, OH, USA) coupled to a gas chromatograph equipped with mass selective detector (Agilent Technologies GC/MS system 7890A/5975C, Santa Clara, CA, USA, with an HP-5 MS capillary column (30 m × 25 μm × 0.25 μm), J&W Scientific, Santa Clara, CA, USA), following the guidelines of USEPA method 5030B. A Lumin (15-2500-074, Teledyne Tekmar, Mason, OH, USA) purge and trap unit with Vocarb 3000 trap (Carbopack B, 10 cm/Carboxen-1000, 6 cm/Carboxen 1001, 1 cm, Supelco, Sigma-Aldrich Co., St. Louis, MO, USA) was used. 5 mL aliquots of water samples were injected into the purging device with a gas-tight syringe. The sample was purged with a stream of helium at a purge flow of 40 mL min^−1^ for 11 min at ambient temperature (20 °C). Desorption by heating the trap was carried out at 250 °C for 2 min. After desorption, the bake temperature was set at 280 °C for 2 min. Chromatographic and MSD conditions were as follows: the inlet temperature was 110 °C and split injection (50:1) was employed. The oven temperature was set at 35 °C (5 min) and raised to 100 °C at 15 °C min^−1^ and ramped up to 225 °C at 25 °C min^−1^ (3 min). Electron ionization was carried out at 70 eV, the ion source temperature was maintained at 230 °C and the transfer line temperature was set at 280 °C. Acquisition was performed in selected ion monitoring/scanning mode from 50–300 amu (SIM/SCAN). The quantitation ion (*m*/*z*) for vinyl chloride was 62 with confirmation ion 64, while for the internal standard fluorobenzene the *m*/*z* were 96 (target ion), and 95 and 97 (qualifier ions). The practical quantitation limit for vinyl chloride was 0.1 µg/L based on the 5 mL of injected samples. The VC removal efficiency (%) was calculated relative to the initial (C_0_) and final (C) concentrations in the treated water samples, as shown in Equation (1):

(1) $$\mathrm{VC}\;\mathrm{removal}\;(\%)=\frac{\left(C_{o}-C\right)}{C_{o}}*100$$

Aldehydes (formaldehyde, acetaldehyde, glyoxal, methylglyoxal) in water were derivatized using O-(2,3,4,5,6-pentafluorobenzyl)-hydroxylamine reagent, extracted with hexane, and further analyzed by GC/MS (USEPA method 556). The practical quantification limits for aldehydes were 0.15–0.80 µg/L [33]. Bromide and bromate concentrations were determined using a Dionex ICS-3000 Ion Chromatography System (Thermo Fisher Scientific, Waltham, MA, USA), following the procedure outlined in DIONEX Application Note 154.

Concentrations of iron and manganese in water samples were determined by flame atomic absorption spectroscopy using a Thermo Scientific™ iCE™ 3000 Series AA Spectrometer, following EPA Method 7000B. Measurements were performed using a flame combination of acetylene and compressed air. The method detection limits and practical quantitation limits were 0.069 mg/L and 0.140 mg/L for Fe, and 0.034 mg/L and 0.069 mg/L for Mn, respectively.

The ozone concentration transferred to the water was determined by iodometric procedure, considering the difference in the input and output ozone concentrations in the gas phase under standard conditions (273 K and 101.3 kPa). Residual ozone was determined in accordance with the standard method, using the indigo trisulfonate reagent [34]. The photon flux (2.3 × 10^−6^ Einsteins s^−1^) and incident light flux (1.5 mW cm^−2^) of UV light were determined by chemical actinometry with potassium ferrioxalate and measured by UVC digital light meter (General Tools & Instruments, Lindenhurst, NY, USA, UV512C).

### 3.5. Artificial Intelligence Modelling of Vinyl Chloride Degradation

All data processing, statistical analysis, feature engineering, and machine learning modeling were performed using Python 3.10 (Python Software Foundation, Wilmington, DE, USA). Data preprocessing and numerical computations were conducted using Pandas 2.1.3 and NumPy 1.26.2 (Open Source Community). Machine learning models were implemented using Scikit-learn 1.4.0 (INRIA, Paris, France). Neural network models were developed using TensorFlow 2.15 (Google LLC, Mountain View, CA, USA). All computations were performed on a workstation running macOS Ventura 13.6 (Apple Inc., Cupertino, CA, USA).

All numerical variables were standardized using z-score scaling prior to model training. The dataset (n = 104) was randomly partitioned into a stratified 70/30 train–test split to preserve the distribution of groundwater and synthetic samples. Hyperparameters for Random Forest, Gradient Boosting, and Ridge models were optimized using grid search with 5-fold cross-validation performed exclusively on the training set. The neural network (13–50–25–1) was trained using the Adam optimizer with early stopping and ReLU activations. A single held-out test set was used for final evaluation to avoid information leakage.

The initial dataset was experimentally obtained under controlled synthetic and groundwater treatment conditions. To increase model robustness, the dataset was augmented using Gaussian perturbation to triple the number of instances while preserving physical constraints and label validity. Input features included raw water quality parameters with the most significant influence on AOP efficiency and process conditions (concentration of iron, manganese, hydrogencarbonate, ammonia nitrogen, TOC, pH, ozone concentration, H_2_O_2_ concentration, UV fluence). The original experimental dataset consisted of 26 unique treatment conditions. After Gaussian perturbation-based augmentation (×3), the final dataset used for model development contained 104 instances. In addition to the original experimental parameters, several engineered features were computed to enhance model expressiveness and reflect known mechanistic relationships relevant to advanced oxidation processes. These derived variables were introduced based on prior domain knowledge and were intended to capture key interactions between chemical species, treatment conditions, and matrix characteristics. Derived variables included the O_3_/H_2_O_2_ ratio, the sum of iron and manganese, the pH/TOC ratio, the O_3_/TOC ratio, and a UV treatment indicator, and are further described in the Supplementary Material. The engineered pH/TOC ratio was included as a domain-informed descriptor reflecting the combined influence of the charge of certain functional groups of organic matter present in groundwater on possible hydroxyl radical scavenging. The inclusion of this ratio was justified by improved model interpretability, as confirmed through permutation importance analysis. These engineered descriptors significantly improved model performance and interpretability, especially by enabling the models to differentiate between treatment regimes and matrix compositions that may otherwise appear similar based on raw input parameters alone.

**Figure 7:**
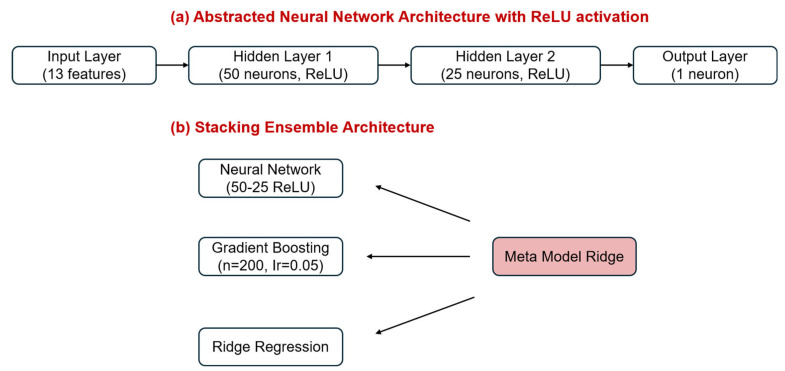
Visualization of AI Model Architectures: (**a**) Abstracted Neural Network Architecture (13-50-25-1) with Rectified Linear Unit (ReLU) activation. The network uses engineered water treatment descriptors and outputs the predicted VC degradation efficiency; (**b**) Stacking Ensemble Architecture. Base learners (Neural Network, Gradient Boosting, and Ridge Regression) are combined via a Ridge meta-regressor to improve generalization across complex treatment interactions.

Multiple regression models were evaluated including: linear regression, Ridge regression (L2 regularization), Random Forest (RF), Gradient Boosting (GB), and a Multi-Layer Perceptron (MLP) [35,36]. The neural network was implemented with an architecture consisting of 13 input nodes, two hidden layers with 50 and 25 neurons, respectively, and a single output neuron. Each hidden layer applied the Rectified Linear Unit (ReLU) activation function (Figure 7a) [37].

To investigate whether the neural network could outperform RF, an ensemble stacking model was implemented. This model combined predictions from three base learners—MLP, GB, and Ridge regression using Ridge as the meta-regressor [38]. The motivation behind stacking was to leverage the complementary strengths of non-linear models and regularized linear estimators for improved generalization (Figure 7b). All models were trained using experimental data obtained from real groundwater samples and synthetic matrices under controlled AOP treatment conditions.

## 4. Conclusions

This study for the first time synergistically integrates the removal of vinyl chloride from real groundwater (11.6–17.8 µg/L) using ozonation and ozone-based AOPs, strengthened by advanced machine learning approaches to accurately model the degradation process. VC degradation by ozonation and ozone-based AOPs is highly dependent on interference from the water matrix, and requires the synergistic use of ozone, hydrogen peroxide and UV light to treat groundwater containing high concentrations of iron, manganese, alkalinity and organic matter. To reduce VC concentrations below 0.5 µg/L, the peroxone process should be applied for GW1, while for GW2, O_3_/H_2_O_2_/UV is required (1.1 mg/L O_3_; 5 mg/L H_2_O_2_; 200 mJ/cm^2^). Oxidative transformation of organic matter leads to higher levels of aldehydes in treated water, with no formation of carcinogenic bromate observed. By combining engineered experimental data with predictive AI models, we achieved high accuracy, with Random Forest performing best. Feature engineering and data augmentation improved robustness across varying groundwater conditions. Neural models were more sensitive to data sparsity, but their integration within ensemble schemes enhanced stability and interpretability. Residual and permutation-importance analyses confirmed ozone dose, pH, and TOC as the dominant drivers of treatment efficiency. Overall, the results show that tree-based and ensemble models offer an interpretable and practical framework for real-time optimization of advanced oxidation processes. While the machine-learning models contributed to interpreting and predicting VC degradation trends, the primary outcomes remain grounded in experimentally validated AOP performance, emphasizing practical relevance for groundwater treatment. Future research should explore extensions to multi-objective modeling, inclusion of reaction by-products, and deployment in hybrid digital-twin systems for full-scale water treatment infrastructure.

## Figures and Tables

**Table 1:** Summary of AI Modelling of VC degradation.

Model	R^2^	MSE	MAE
Random Forest	0.987	10.78	2.488
Gradient Boosting	0.982	14.97	2.796
Stacked Ensemble (NN + GB + Ridge)	0.981	15.73	2.390
Linear Regression	0.956	36.20	4.535
Ridge Regression	0.953	38.89	4.252
Neural Network (MLP)	0.899	82.97	6.194

R^2^: the proportion of variance explained by the model; MSE: the average of the squared differences between predicted and actual values; MAE: the average of the absolute differences between predicted and actual values.

**Table 2:** Physical–chemical characteristics of the raw groundwater (presented standard deviations of n = 10 measurements).

Parameter	Unit of Measurement	GW1	GW2
pH	-	7.14 ± 0.17	6.83 ± 0.25
Electrical conductivity	µS/cm	1317 ± 136	1652 ± 52
Turbidity	NTU	59.9 ± 11.3	68.7 ± 9.3
Total organic carbon (TOC)	mg/L C	4.05 ± 1.6	4.35 ± 1.6
Total aldehydes	µg/L	4.30 ± 1.65	6.32 ± 1.55
Formaldehyde	µg/L	2.50 ± 0.55	3.81 ± 0.73
Acetaldehyde	µg/L	1.30 ± 0.31	1.93 ± 0.22
Glyoxal	µg/L	0.32 ± 0.18	0.38 ± 0.15
Methylglyoxal	µg/L	0.18 ± 0.05	0.20 ± 0.10
Permanganate index	mg/L	12.6 ± 0.25	14.2 ± 0.15
Ammonia	mg N/L	1.10 ± 0.33	3.30 ± 1.18
Nitrates	mg N/L	0.21 ± 0.09	0.24 ± 0.06
Nitrites	mg N/L	0.02 ± 0.01	0.04 ± 0.01
Orthophosphates	mg P/L	0.24 ± 0.13	0.26 ± 0.10
Hydrogencarbonates	mg/L	676 ± 35	788 ± 25
Bromide	mg/l	0.02 ± 0.03	0.03 ± 0.1
Hardness	mg/L	494 ± 120	700 ± 56
Iron	mg/L	6.06 ± 2.2	16.5 ± 2.1
Manganese	mg/L	0.26 ± 0.06	0.3 ± 0.06
Vinyl chloride	µg/L	11.6 ± 0.61	17.8 ± 0.74

## Data Availability

The data presented in this study are available on request from the corresponding author.

## Supplementary Materials

The following supporting information can be downloaded at: https://www.mdpi.com/article/10.3390/molecules30244737/s1, Figure S1: Removal of VC from synthetic and groundwater using ozonation; Figure S2. The schematic diagram of the water treatment units; Table S1: Water characteristics after oxidation treatments.
