# Supplementary material for: Vinyl Chloride Degradation Using Ozone-Based Advanced Oxidation Processes: Bridging Groundwater Treatment and Machine Learning for Smarter Solutions
Source: Molecules. 2025 Dec 11;30(24):4737. doi: 10.3390/molecules30244737 (PMC12736060; doi:10.3390/molecules30244737)
Supplement: Supplementary file 1 [file molecules-30-04737-s001.zip › molecules-4010312-supplementary.pdf]

Supplementary material

Vinyl Chloride Degradation Using Ozone-Based Advanced Oxidation Processes: Bridging Groundwater Treatment and Machine Learning for Smarter Solutions

Jelena Molnar Jazić[1]*, Marko Arsenović[2]*, Tajana Simetić[1], Slaven Tenodi[1], Marijana Kragulj Isakovski[1], Aleksandra Tubić[1], Jasmina Agbaba[1]

[1]Department of Chemistry, Biochemistry and Environmental Protection, Faculty of Sciences, University of Novi Sad, Trg Dositeja Obradovića 3, 21000 Novi Sad, Serbia
[2]Faculty of Technical Sciences, University of Novi Sad, Trg Dositeja Obradovića 6, 21000 Novi Sad, Serbia

jelena.molnar@dh.uns.ac.rs; arsenovic@uns.ac.rs

S2. Results and Discussion

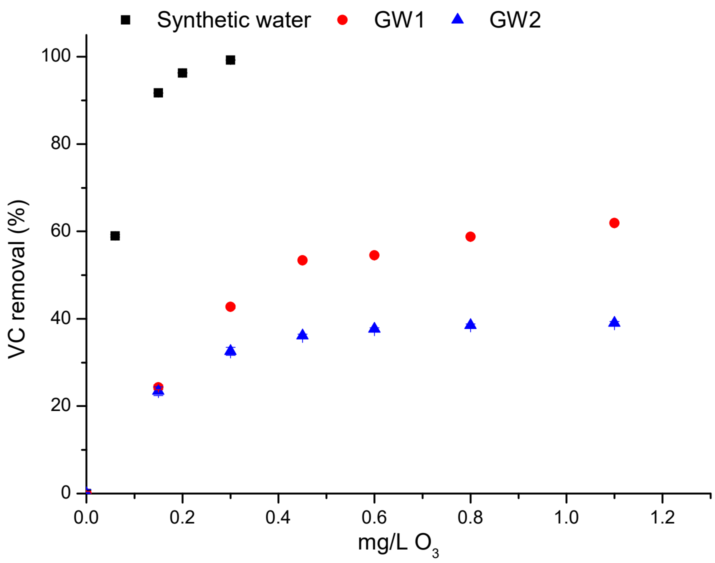

Figure S1. Removal of VC from synthetic and groundwater using ozonation

Table S1. Water characteristics after oxidation treatments

Parameter	Unit of measurement	Treatment			
		O_3[1]	O_3/H_2O_2[2]	O_3/UV[3]	O_3/H_2O_2/UV[4]
		GW1			
TOC	mg/L	3.15±0.6	2.95±0.5	2.90±0.55	2.90±0.35
Iron	mg/L	<0.10	<0.10	<0.10	<0.10
Manganese	mg/L	0.12±0.02	0.12±0.03	0.12±0.02	0.11±0.02
Formaldehyde	µg/L	13.5±0.32	18.3±0.04	14.3±0.68	14.1±0.47
Acetaldehyde	µg/L	12.8±0.15	15.5±0.21	13.5±0.57	12.1±0.35
Glyoxal	µg/L	11.3±0.18	13.5±0.98	11.8±0.77	13.5±0.98
Methylglyoxal	µg/L	8.12±0.23	9.01±0.75	8.97±0.14	11.2±0.76

Parameter	Unit of measurement	Treatment			
		O_3[1]	O_3/H_2O_2[2]	O_3/UV[3]	O_3/H_2O_2/UV[4]
Total aldehydes	µg/L	45.7±1.95	56.3±2.66	48.6±2.47	50.9±2.98
Bromate	µg/L	<10	<10	<10	<10
		GW2			
TOC	mg/L	3.25±0.3	3.10±0.25	3.05±0.15	3.05±0.15
Iron	mg/L	0.70±0.05	0.60±0.22	0.70±0.1	0.70±0.05
Manganese	mg/L	0.22±0.02	0.21±0.03	0.21±0.03	0.21±0.03
Formaldehyde	µg/L	18.9±1.53	21.5±3.58	19.1±1.51	20.5±1.87
Acetaldehyde	µg/L	13.6±2.50	17.3±2.01	14.5±2.41	15.5±2.06
Glyoxal	µg/L	11.5±0.45	14.5±1.43	12.1±0.38	13.5±0.78
Methylglyoxal	µg/L	10.2±1.63	9.87±3.01	10.5±1.32	10.7±1.09
Total aldehydes	µg/L	54.2±3.05	63.2±6.02	56.2±1.87	60.2±5.47
Bromate	µg/L	<10	<10	<10	<10

[1] 1.1 mg/L O_3;
[2] 1.1 mg/L O_3; 1 mg/L H_2O_2;
[3] 1.1 mg/L O_3; 200 mJ/cm^2;
[4] 1.1 mg/L O_3; 1 mg/L H_2O_2; 200 mJ/cm^2.

A complex reaction chain mechanism of peroxone process and UV-based AOPs:

$H_2O_2 \rightarrow HO_2^- + H^+$ 1

$HO_2^- + O_3 \rightarrow HO_2^{\cdot} + O_3^{\cdot -}$ 2

$O_3^{\cdot -} + H^+ \rightarrow HO_3^{\cdot -}$ 3

$HO_3^{\cdot -} \rightarrow O_2 + HO^{\cdot}$ 4

$HO^{\cdot} + H_2O_2 \rightarrow HO_2^{\cdot} + H_2O$ 5

$O_3 + H_2O \xrightarrow{h\nu} H_2O_2 + O_2$ 6

$O_3 + H_2O_2 \rightarrow HO_2^{\cdot} + HO^{\cdot} + O_2$ 7

$H_2O_2 \xrightarrow{h\nu} 2HO^{\cdot}$ 8

$H_2O_2 + H_2O \rightleftarrows H_3O^+ + HO_2^-$ 9

S3. Materials and methods

S3.3. Ozonation and ozone-based AOPs

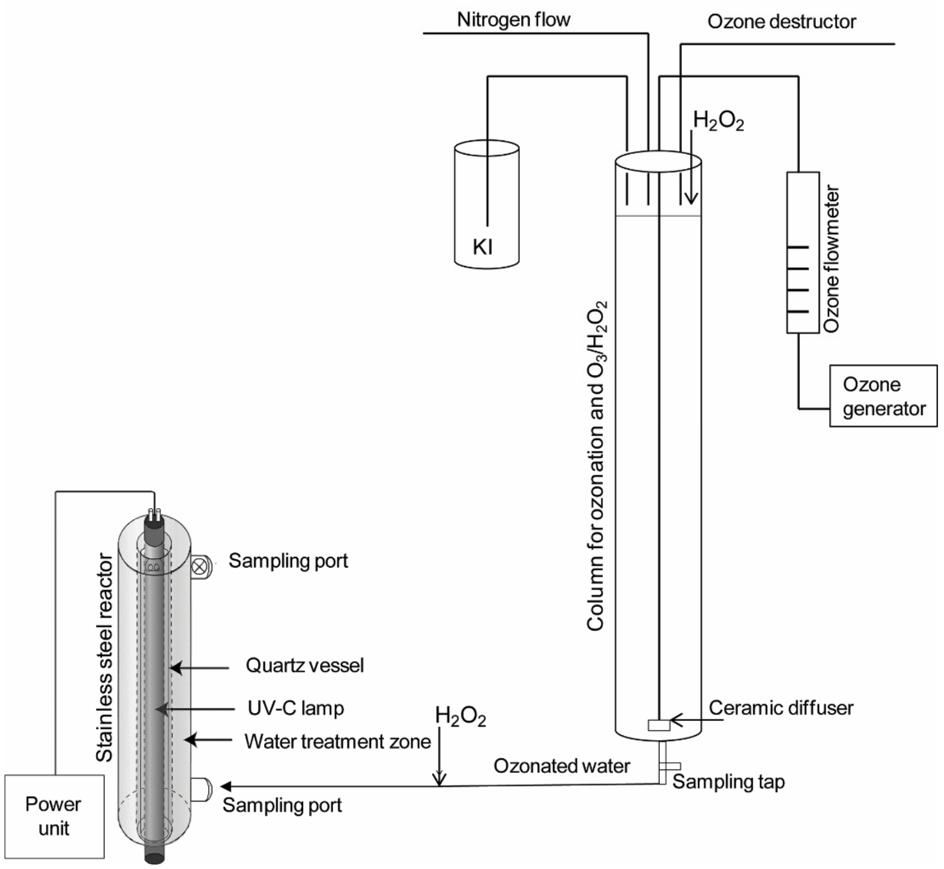

Figure S2. The schematic diagram of the water treatment units

S3.4. Analytical methods

Permanganate index: SRPS EN ISO 8467:2007 (Water quality - Determination of permanganate index).

Ammonia: SRPS H.Z1.184: 1974 (Water testing - Determination of ammonia).

Nitrates: SRPS ISO 7890-3:1994 (Water quality — Determination of nitrate — Part 3: Spectrometric method using sulfosalicylic acid).

Nitrites: SRPS EN 26777:2009 (Water quality - Determination of nitrite - Molecular absorption spectrometric method).

Orthophosphates: SRPS EN ISO 6878:2008 (Water quality - Determination of phosphorus - Ammonium molybdate spectrometric method).

Hydrogencarbonates: SRPS EN IS 9963-1:2007 (Water quality - Determination of alkalinity - Part 1: Determination of total and composite alkalinity).

Hardness: 2340 C (Standard Methods for the Examination of Water and Wastewater, 24th ed., APHA-AWWA-WEF, 2022).

Turbidity: 2130 B (Standard Methods for the Examination of Water and Wastewater, 24th ed., APHA-AWWA-WEF, 2022).
TOC content was analysed in water samples by Elementar LiquiTOCII, with oxidation by platinum catalysed combustion at 850 °C in accordance with method (SRPS ISO 8245:2007; Water quality - Guidelines for the determination of total organic carbon (TOC) and dissolved organic carbon (DOC)).
The pH was measured by a portable instrument (WTW InoLab pH) - SRPS H.Z1.111: 1987 (Testing of industrial waters - Measurement of pH - Potentiometric method).

S3.5. Artificial Intelligence Modelling of Vinyl Chloride Degradation

The supplementary section provides additional methodological detail regarding engineered features, data processing, and model development referenced in the main text.

Engineered Input Variables
Full List of Input Features Used in Machine Learning Models
To ensure clarity and reproducibility, the complete set of input variables used for model development is provided below. The feature set consists of:
(1) Raw experimental variables:
- pH
- Total organic carbon
- Iron
- Manganese
- Hydrogencarbonates
- Ammonia nitrogen
- Ozone concentration
- Hydrogen peroxide concentration
- UV fluence
- Treatment type indicator (ozonation / peroxone / O_3/UV / O_3/H_2O_2/UV)

(2) Engineered features derived from chemical and operational relationships:
- O_3/H_2O_2 ratio
- Fe + Mn sum
- pH/TOC ratio
- O_3 per TOC (O_3/TOC)
- UV treatment indicator (has_uv)

These variables jointly represent the physicochemical water-matrix characteristics and oxidation process conditions that influence vinyl chloride degradation kinetics.

To enhance model interpretability and predictive capacity, several engineered features were computed based on chemical and operational logic. These include:
O_3/H_2O_2 ratio (*O3_H2O2_ratio*) – calculated as the ratio between the applied ozone and hydrogen peroxide doses. This variable approximates the stoichiometric balance in peroxone-based systems and was computed as:

$$O_3\_H_2O_2\_ratio = O_3 / (H_2O_2 + \varepsilon)$$

Fe + Mn sum (*Fe_Mn_sum*) – representing the combined concentration of iron and manganese as trace metals, which are known to influence radical generation in water matrices:

$Fe\_Mn\_sum = Fe + Mn$

pH/TOC ratio (*pH_TOC_ratio*) – design to capture the influence of the charge of certain functional groups of organic matter present in groundwater on treatment efficiency depending on pH variation, defined as:

$pH\_TOC\_ratio = pH / (TOC + \varepsilon)$

O_3 per TOC (*O_3_per_TOC)* – expressing ozone dose normalized to organic carbon concentration, used as a indicator for oxidative impact on the matrix:

$O_3\_per\_TOC = O_3 / (TOC + \varepsilon)$

UV treatment indicator (*has_uv*) – a binary variable engineered to represent the presence or absence of UV irradiation, which was not explicitly categorized in the raw dataset. This variable was defined based on UV fluence as follows:

- has_uv = 1 if UV fluence > 0;
- 0 otherwise

In all cases, $\varepsilon = 10^{-6}$ was used as a small constant to avoid division by zero.

Data Augmentation Strategy

To address limited data size and enhance generalizability, synthetic samples were generated by applying Gaussian noise (5% standard deviation) to original numeric records. This expanded the dataset threefold, preserving the original feature distribution.

Model Training and Evaluation

All models were implemented in Python using scikit-learn and keras. Ensemble learning methods (Random Forest, Gradient Boosting) and neural networks (MLP) were trained using a 70:30 train-test split. Hyperparameter optimization was conducted via randomized search. Evaluation metrics included R^2, MSE, and MAE.

Residual and Feature Importance Analysis

Permutation feature importance was used to assess model interpretability. Feature mappings and relevance are shown in Figure 7 of the main manuscript. Residuals and distribution plots are available upon request.

Standards and Compliance

Experimental work and sample analysis followed ISO 17025 and APHA-AWWA-WEF 2022 guidelines for water quality monitoring.

References

Rekhate, C.; Srivastava, J.K. Recent advances in ozone-based advanced oxidation processes for treatment of wastewater - A review. Chem. Eng. J. Adv. 2020, 3, 100031. https://doi.org/10.1016/j.ceja.2020.100031.
